# Influence of selant fibrin on the wound healing of the pigs vocal folds

**DOI:** 10.1590/S1808-86942012000100008

**Published:** 2015-10-20

**Authors:** Karla Palma Portes, André de Campos Duprat, Carmen Lucia Penteado Lancellotti, Leonardo Silva, Flávia Coelho de Souza

**Affiliations:** a Specialist in otorhinolaryngology, ABORL-CCF (Master's degree student, Medical Science School, São Paulo Holy House (Santa Casa); b Doctoral degree in otorhinolaryngology (Professor/Instructor, Medical Science School, São Paulo Holy House (Santa Casa); c Doctoral degree in Pathology (Full professor, Medical Science School, São Paulo Holy House (Santa Casa); d Doctoral degree in otorhinolaryngology (Professor/Instructor, Medical Science School, São Paulo Holy House (Santa Casa); e Master's degree in veterinary medicine (Doctoral student, Sao Paulo Holy House (Santa Casa). Medical Science School, São Paulo Holy House (Santa Casa). Advanced Science Institute in Otorhinolaryngology

**Keywords:** fibrin tissue adhesive, vocal cords, wound healing

## Abstract

Fibrin sealants or fibrin glue are products made from human plasma proteins, which mimic the final pathway of the coagulation cascade. Its application to stimulate the healing process has been a topic of debate in the literature. The use of fibrin sealants in phonosurgery has been empirical; there have been no studies that investigate the action of fibrin sealant in Reinke's space.

**Aim:**

To evaluate the effect of fibrin glue in healing of the vocal folds of pigs after surgical manipulation.

**Materials and Methods:**

This was a prospective and experimental study. Six animals had both vocal folds incised. Sealant was applied in one of them; the other served as a control. After three months, the animals were sacrificed and a collagen count was carried out.

**Results:**

The side on which glue was applied had an average of 27.8% against 20.4% of the side without glue.

**Conclusion:**

The collagen concentration in the samples where the fibrin sealant was applied was significantly higher compared to samples without glue. Thus, the presence of a fibrin sealant stimulates fibrogenesis in this tissue.

## INTRODUCTION

Fibrin sealants or fibrin glue are products made from human plasma proteins; they mimic the final pathway of the coagulation cascade. In these products, fibrinogen is cleaved by proteolysis and converted into fibrin polymers by the effect of thrombin. Factor XIII reacts with fibrin polymers when activated by thrombin in the presence of calcium ions, yielding a stable and insoluble clot^1^, ^2^.

The FDA in the United States has approved fibrin glue for use during surgery because of its hemostatic properties. Fibrin glue has also been applied in several surgical procedures as a sealant of cavities, stimulator of tissue repair, and as a slow-release agent for drugs or growth factors^3^, ^4^, ^5^, ^6^, ^7^, ^8^.

There are several debates in the medical literature about the use of fibrin sealants to stimulate healing. Clark^9^ has stated that using fibrin sealants to avoid adhesions or fibrosis is contradictory because fibrinogen stimulates production of collagen. However, the majority of experimental clinical studies on the use of fibrin sealants in several tissues have yielded good results; in these studies, fibrin glue reduces fibrosis and tissue adhesion in different surgical fields compared to controls^10^.

Bouchayer & Cornut^11^ (1992) were the first to report using fibrin sealants in laryngeal surgery. Sealants are generally used to fix mucosal flaps^12^, to serve as a base for placing grafts in Reinke's space^13^, and mostly – after the advent of CO_2_ laser – to cover a surgical field thereby avoiding formation of granulomas^14^.

Fibrin sealants have been used empirically in phonosurgery. No papers have been published on the effect of fibrin sealants in Reinke's space or of their direct effect on fibrinogenesis during healing. The main purpose of this study was to assess the effect of fibrin glue on the healing process after surgical handling of pig vocal folds.

## MATERIALS AND METHODS

**Figure 1:**
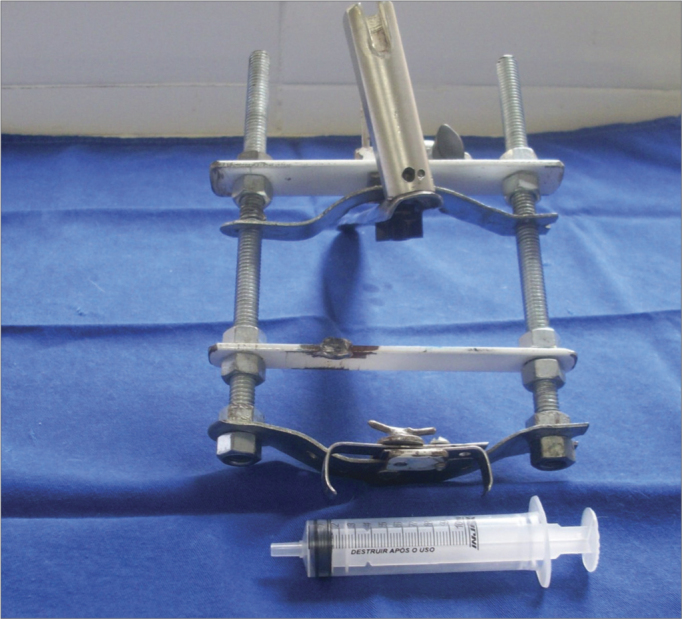
Mouth opener specifically developed for laryngoscopy in pigs (Pontes et al, 2007).

**Figure 2:**
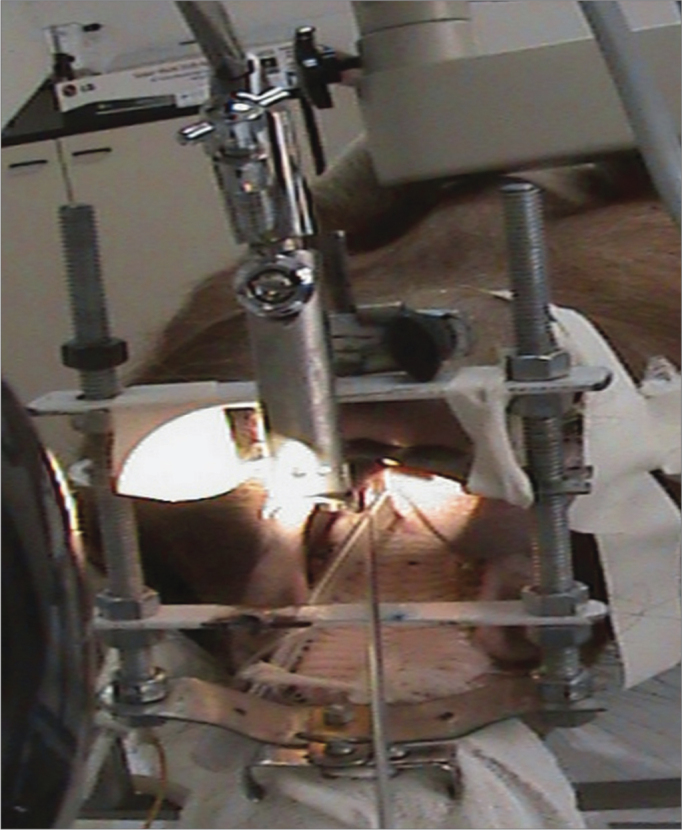
View by using the mouth opener for carrying out the procedure.

The study was started after approval by the institutional review board (protocol 2/08). Six pigs (mini pigs) were required for this study. These animals were monitored and anesthetized with orotracheal intubation, after which they were placed in inclined dorsal decubitus^15^. A suspension laryngoscope specifically developed for procedures in pigs (Pontes et al.) was used for visualizing the larynx (Figures 1 and 2)^16^.

**Figure 3:**
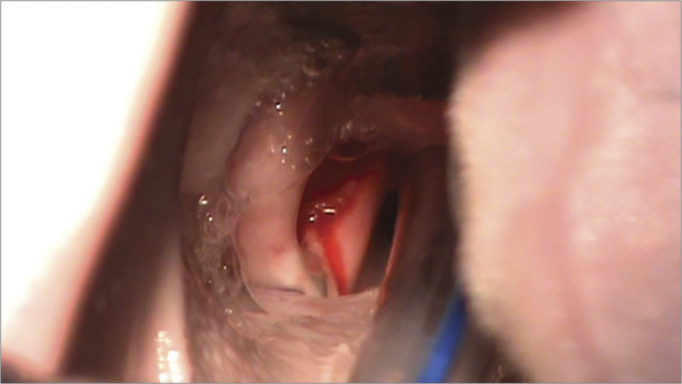
Incision on the vocal fold.

A straight binocular surgical microscope with a 400 mm lens was used for observing the glottis and carrying out the procedure. An incision was made on both vocal folds and the mucosa was detached (Figure 3) carefully not to damage the thyroarytenoid muscle. Next, about 0.5 ml of the fibrin sealant was applied into the pouch that was made from one of the vocal folds, over which the mucosa was placed. The mucosa was placed without sealant over the other vocal fold for second intention healing. The vocal fold on which sealant was placed was chosen randomly. The commercial sealant used in this study was Tissucol^®^ (Baxter laboratories); it contains 70-110 mg/ml fibrinogen, 10-50 U/ml human factor XIII, 500 UI/ml human thrombin, and 3000 KIU bovine aprotinine. The packages contain syringes and needles to reconstitute the product and a Duploject administration device.

The flasks in the kit were heated at a constant temperature (37°C) in a thermal container until the procedure was done. Freeze dried Tissucol was reconstituted in an aprotinine solution. Freeze dried thrombin was reconstituted in a calcium chloride solution. Quick solidification (<20 seconds) requires thrombin (500 U), and slow clotting (40-60 seconds) requires thrombin (4 U). We chose quick solidification thrombin. The two components were applied simultaneously in equal quantities by using the Duploject^®^.

The animals remained in a sty for three months after surgery and then were sacrificed by using intramuscular 0.1 mg/Kg *Dormonid (*1mg/ml) and endovenous 6 mg/kg propofol, followed by an intracardiac potassium chloride injection^15^.

After the animals had been sacrificed the larynx was removed and placed in 10% formaldehyde. Each larynx was sectioned in two hemilarynxes for histology. The histology slides were prepared for both specimens and stained Picrosirius Red^17^. The slides were assessed under optic microscopy to search for anomalies such as foreign bodies or remains of the fibrin sealant. A video camera was attached to the optic microscope. Images were analyzed using the *Image-Pro Plus* 4.5 software for *Windows* in a computer with a *Pentium IV*^®^ processor.

Fibroplasia was measured as square micrometers (μm^2^). Three specific areas of the mucosa were chosen using the computer mouse. The picrosirius red stained collagen was automatically selected, quantified, and its area given in μm^2^ in the *Image-Pro Plus* 4.5 software. At the end of the randomization period, data were transported to the Excel for Window^®^ software and tabulated for the statistical analysis, which was done using the SPSS V16, Minitab 15, and Excel Office 2007 software.

## RESULTS

**Figure 4:**
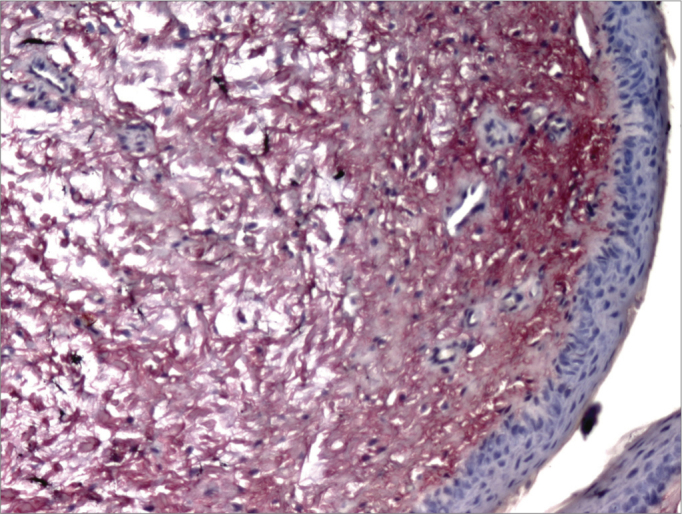
PVD pig 76 – Fibrin sealant was applied to this vocal fold; note intense fibroplasia (stained red)

**Figure 5:**
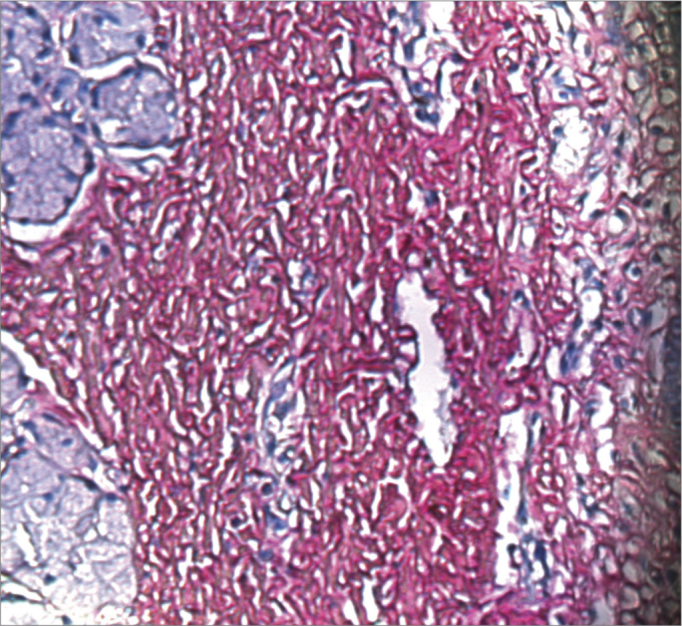
PVD Pig 79 – Note the intense red; collagen fibers are stained by picrosirius red, and contrast with the epithelial and gland tissues (blue).

Picrosirius Red stained the collagen layers in red, which made it easier for the computer software to measure its concentration (Figures 4 and 5). No specimen contained remains of the fibrin sealant. The collagen count was done using a video camera coupled to an optical microscope. There were two picrosirius red stained slides for each animal, corresponding to the right and left sides. Three fields in each slide were photographed.

**Chart 1:**
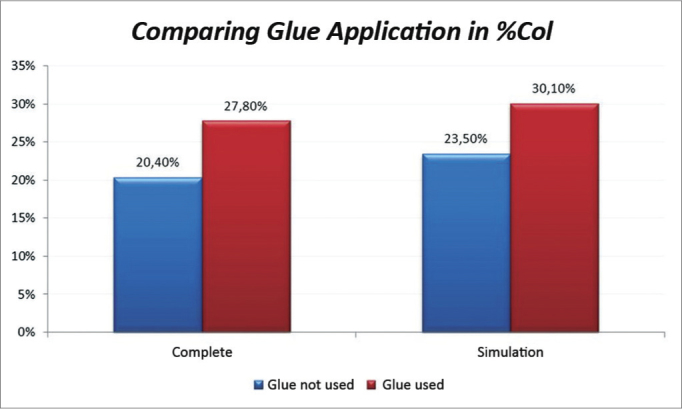
Comparison of collagen concentrations among vocal folds with and without glue; analysis of the Complete and Simulation groups.

The significance level for this study was 0.05 (5%). The confidence intervals were based on a 95% statistical confidence level. The Wilcoxon test was used for the statistical analysis of paired data, as each subject was its own control. We immediately found that the measures in the left of pig 77 and in the right of pig 79 were outliers (very high values); thus, the analysis was done in two steps. The entire sample was named *Complete*, and the sample without the pigs 77 and 79 was named *Simulation*. Table 1 shows the results of the Wilcoxon test; we used the results of the positional description to assess the collagen concentrations in the study areas. The difference was considered statistically significant in the *Complete* sample; in this case, the mean in the side in which glue was applied was 27.8% compared to 20.4% in the side without glue. (Chart 1).

**Table 1:** Comparison of application of glue in % of collagen.

% Collagen	Complete		Simulation	
	No Glue	Glue	No Glue	Glue
Mean	20.4%	27.8%	23.5%	30.1%
Median	16.7%	23.4%	20.9%	30.5%
Standard deviation	9.3%	9.4%	9.8%	10.8%
Q1	14.8%	22.2%	14.9%	21.6%
Q3	22.4%	35.8%	31.0%	39.6%
N	18	18	12	12
CI	4.3%	4.3%	5.5%	6.1%

*p*-value	0.002		0.060

## DISCUSSION

A first analysis of the slides did not reveal remains of the fibrin sealant in any sample, which suggests that it was fully absorbed at a later phase in healing. One of the most significant advantages of the fibrin sealant is clot stability in the sealed area, which is determined by antifibrinolytic agents in the glue; this stops displacements in the sealed area and its full resorptive properties avoids delays in healing^18^. Aprotinine, a bovine antifibrinolytic agent in several commercial products, is responsible for clot stability and prolonged duration in a surgical site^19^. The ideal concentration of aprotinine is 3000 KIU/ml^18^.

Analysis of the fibroplasty showed a higher collagen concentration in vocal folds where glue was applied, compared to the contralateral vocal fold (control). The difference was statistically significant (27.8% against 20.4% in the side without glue). The difference was found even in pigs 76 and 79, which were considered outliers. These samples were outside the range of others possibly because of time differences and variations in the stains used at different times. No animal presented signs of infection that could affect healing throughout the observation period. This study compares vocal folds within the same animal; thus, possible differences in the formula of stains did not affect the comparative analysis.

The medical literature contains many studies showing the efficacy of healing after fibrin sealants are applied. Papers describing the use of fibrin sealants in laryngeal surgery – and especially phonosurgery – are case reports. Sealants have been applied empirically and the functional results are assessed postoperatively. There are no studies describing the effect of fibrin sealants on the healing of the lamina propria from the perspective of histology. Our results were compared with those in other studies in different tissues and animals.

We found a few studies describing more intense inflammation in the surgical sites within the first few weeks after fibrin sealants were applied; this process may persist for up to 30 days after application. Chronic inflammation develops and a fibroblast-rich granulation tissue forms. This persistent fibroblast-rich infiltrate suggests that a collagen-rich healing process may develop in the long term, and consequently more fibrosis^20^. Hanson & Quinn^21^ carried out a study to demonstrate the effect of fibrin glue on neutrophil chemotaxis in healing. These authors found that the fibrin concentration, which varies among commercially available fibrin sealants, has a direct effect on chemotaxis. Higher fibrin concentrations are associated with less chemotaxis; concentrations over 2mg/ ml may block the process. The authors also believe that the presence of factor XIII, which is found in some commercial sealants, may affect the process directly, thereby increasing neutrophil chemotaxis and explaining why there is more inflammation in the presence of fibrin glue and more fibroplasty at later phases in healing.

Clark^9^ reviewed the literature on the action of fibrin sealants in tissue repair and argues that their use as tissue healing promoters and preventers of abdominal adhesion appears contradictory; an agent that promotes tissue repair may increase tissue adhesion rather than prevent it. There is also the study published by Marx & Mou^22^. These authors reported intense inflammation in the dermis up to the fourth day, which subsided by the seventh day at which point there was full reepithelization, marked fibroplasia, collagen deposits, and angiogenesis along the surgical wound.

Fabris et al.^23^ (1998) studied the effect of highly concentrated fibrin sealants on the growth of fibroblasts in the human periodontal ligament. These fibroblasts were cultured for 48 h and 72 h in the presence of fibrin glue; the results showed that it is compatible with fibroblast growth and collagen synthesis, although the authors noted that there was a trend towards lower cell proliferation and collagen production compared to controls.

An important issue was the likelihood of an immune reaction to the glue in our sample. The components of fibrin sealants are of human origin and aprotinine is of bovine origin. The preparation method of current sealants reduces significantly the possibility of anaphylaxis (by aprotinine) in humans, but this possibility may not be excluded because of phylogenetic differences among species. Scardino et al.^24^ made a histological analysis of epithelial healing under the effect of two different sealants – a human plasma derived product and a product derived from bovine plasma. The study sample consisted of 24 female beagle dogs. The authors showed that there was mild to moderate chronic inflammation in both groups; inflammation persisted more in the group where the bovine sealant was used, probably because of the phylogenetic difference between the commercial products and the animal model chosen for the study.

Studies assessing directly the effect of fibrin sealants on tissue healing show discrepant results in the medical literature. On the other hand, case studies almost unanimously report good functional and esthetic results when sealants are used. Thus, surgeons should exert care and weight the costs and benefits of sealants in their daily practice.

In the present study, incisions on the vocal folds of pigs generated small open areas and insignificant bleeding. In such cases, the cost of fibrin sealants and results that suggest increased collagen deposition in healed tissues make their use disadvantageous compared to simply approximating the borders of the wound. Conversely, when there is a need to fix surgical flaps, extensive open and widely dissected areas, or when the overlying tissue to the vocal folds is disorganized, approximating the borders of the wound with fibrin sealants and stimulation of healing becomes beneficial. Our study touches on topics of great concern for phonosurgeons: to understand the intricate healing process of the lamina propria, and to find substances that may minimize surgical aggression to this delicate tissue. We have little data to state whether fibrin sealants are essential or not for vocal fold regeneration, as well as the degree to which they generate fibrosis and adhesions that may affect voice. There are few published studies about the action of fibrin glue on the lamina propria; we know that this effect cannot be seen as similar to that on epithelial or vascular cells.

Advances in biomolecular medicine and the possibility of culturing human cells in vitro may make it possible to understand in greater detail the behavior of human cells in the presence of fibrin sealants without phylogenetic interferences that permeate studies using animals.

## CONCLUSION

The following conclusions can be made based on the present study:

- We found no fibrin sealant in the study samples, which confirms that this substance is fully absorbed by the body;
- The concentration of collagen in samples where the fibrin sealant was applied is significantly higher compared to the sealant-free samples. Thus, it stimulates fibrinogenesis in this tissue.
